# Supplementary material for: Primary molt in Gruiforms and simpler molt summary tables
Source: PeerJ. 2018 Sep 7;6:e5499. doi: 10.7717/peerj.5499 (PMC6130233; doi:10.7717/peerj.5499)
Supplement: Supplemental Information 1 [file peerj-06-5499-s001.docx]

We used the following codes in our molt tables; some codes are only relevant to flufftails and rails, where molt data was summarized from Stresemann and Stresemann (1966):

t = terminal feather

n = nodal feather

pp = directionality score of proximal between two feathers where one feather is a stable node

dd = directionality score of distal between two feathers where one feather is a stable node

p = proximal molt between growing feathers

d = distal molt between growing feathers

? = indicates uncertainty, used for directionality scores and nodal/terminal feathers.

sab = sheathing at base; indicates a full grown feather that just completed molt

0 = old feather

1 = new feather

.1-.99 = indicate growing feathers and estimates the length of the growing feather (.9=90% full length)

m = missing

0.01 = also missing

J = juvenile feather

0/J = indicates either old (0) or juvenile (J) feather

0/1 = uncertainty in feather age (0=old; 1=new)

ND = no data

pres = feather present but no age assigned by the Stresemanns

“very pretty”= direct transcription of the Stresemanns’ data and presumably indicates a newly replaced feather

g = growing feather (see below for interpreting growing feathers scored by the

Stresemanns)

Below, we provide background information for our interpretations of the Stresemanns’ molt data on rails and flufftails.

Stresemann and Stresemann (1966) estimated lengths of growing feathers in multiple ways: some pin-feather lengths were estimated in cm; some received estimates of the length of feather that remained to be grown; for others, the length of the growing feather was estimated as a fraction of its full length. In the latter case we converted these estimates to decimal fractions.

The Stresemanns commented that in many forest rails it was difficult to distinguish new and old feathers, so they noted ambiguity about feather age in carious descriptions. We have put these descriptions in the notes column, and either assigned no age or their apparent best guess in the column for each primary.

Our scoring of the Stresemann’s data is as follows: 1 = new; 0 = old. For growing feathers, the Stresemanns always indicated growing before a length estimate, so we list these growing primaries as g, followed by their length measure. Thus, g 1/2 means half grown; g 2cm means a 2cm long feather. In some cases they say pin, and give a length, which we so transcribe. They also use growing and give how much of the feathers is not yet grown ("fehlen" 2cm), which we coded as "g -2cm". We interpreted "noch w" or "still growing" as a nearly full length feather, possibly with sheathing at its base, though sheathing is sometimes directly noted, which we recorded as sab for "sheathing at base".

When ages are unclear the Stresemanns used "stehen" for feathers that are present (standing) but not assigned to age; we transcribed this as “pres” for present, but provide no age assignment.

The Stresemanns’ directionality scores of descendent are defined on page 11 of their monograph and equate to proximal replacement.

Nodal and terminal follow the usual definition, but we assume P10 and P1 represent start or end points for molt; for most specimens there is no data on S molt. P10 and P9 were often scored as fractions of full, which creates an issue because we do not know their full lengths, making it impossible in many cases to know which was lost first. Further, they are often lost in quick succession, as is true of many species of passerines and shorebirds with rapid dropping of the inner most primaries.

As for other species we examine, we do not score direction for nodes and termini between P10 and P1, but we do score direction between P10 and P9 and between P2 and P1, but record these scores as pp or dd to indicate that they can be considered directions IF these feathers turn out to be stable nodes or termini.

Because growing feathers often have neighbors that were not aged (the Stresemanns used the term standing, which we transcribed as present), nodes and termini often could not be scored. The Stresemanns were quite explicit about the difficulty of aging feathers in these rails.

Feather loss is also rapid enough in the species that loose feathers in quick succession that direction often cannot be deduced from the length information given by the Stresemanns between adjacent growing feathers. But, other times, they say specifically which was longer; for these we assign direction. When direction cannot be inferred between adjacent growing feathers we assigned a "?". This occurs when they are the same fractional or measured length AND when their neighbor was not aged, but only recorded as present. O? and N? are treated as old and new because these seemed to be the Stresemanns’ best guess.
